# Integrative Evaluation of Automated Massage Combined with Thermotherapy: Physical, Physiological, and Psychological Viewpoints

**DOI:** 10.1155/2016/2826905

**Published:** 2016-12-15

**Authors:** Do-Won Kim, Dae Woon Lee, Joergen Schreiber, Chang-Hwan Im, Hansung Kim

**Affiliations:** ^1^Department of Biomedical Engineering, Chonnam National University, 50 Daehak-ro, Yeosu-si 59626, Republic of Korea; ^2^Berlin Institute of Technology, Machine Learning Group, Marchstraße 23, 10587 Berlin, Germany; ^3^Yonsei Fraunhofer IZFP Medical Device Lab, Yonsei University, Medical Industry Techo Tower 205, Wonju 220-710, Republic of Korea; ^4^Fraunhofer Institute for Ceramic Technologies and Systems, Maria-Reiche-Straße 2, 01109 Dresden, Germany; ^5^Department of Biomedical Engineering, Hanyang University, 17 Haengdang-dong, Seongdong-gu, Seoul 133-791, Republic of Korea; ^6^Department of Biomedical Engineering, Yonsei University, Medical Industry Techno Tower 307, Wonju 220-710, Republic of Korea

## Abstract

Various types of massages are reported to relieve stress, pain, and anxiety which are beneficial for rehabilitation; however, more comprehensive studies are needed to understand the mechanism of massage therapy. In this study, we investigated the effect of massage therapy, alone or in combination with infrared heating, on 3 different aspects: physical, physiological, and psychological. Twenty-eight healthy university students were subjected to 3 different treatment conditions on separate days, one condition per day: control, massage only, or massage with infrared heating. Physical (trunk extension [TE]; maximum power of erector spinae), physiological (heart-rate variability [HRV]; electroencephalogram [EEG]), and psychological (state-trait anxiety inventory [STAI]; visual analogue scale [VAS]) measurements were evaluated and recorded before and after each treatment condition. The results showed that massage therapy, especially when combined with infrared heating, significantly improved physical functioning, increased parasympathetic response, and decreased psychological stress and anxiety. In the current study, we observed that massage therapy contributes to various physical, physiological, and psychological changes, where the effect increases with thermotherapy.

## 1. Introduction

Massage therapy is a systematic manipulation of soft tissues with rhythmical pressure and stroking which contributes to relieving various types of body distress [1, 2]. Massage therapy is often used as a complimentary therapy to support pharmacological treatment with sedatives and analgesics for reducing stress, pain, or anxiety of the patients. However, controlled studies investigating the mechanisms underlying the benefits of massage therapy were absent until recently [3].

Over the past two decades, investigators have shown the effects of massage therapy on various physiological features, such as blood pressure [4, 5], heart-rate variability (HRV) [6], and electroencephalogram (EEG) [3, 7], and also in terms of psychological abilities, such as mental operations and psychological record [8–11]. In most studies, researchers have reported that massage therapy relieves psychological or physiological stress, for instance, chronic pain (e.g., headache and low back pain) [12–14], muscle fatigue, anxiety [15, 16], or depression [7, 17, 18]. Therefore, massage therapy is often used for rehabilitation purposes, often in combination with other rehabilitation methods to maximize the effect.

However, despite various attempts to find the underlying mechanism of massage therapy, it is still unclear how exactly massage therapy affects our body. Researchers suggested that massage therapy activates the parasympathetic nervous system, which in turn decreases blood pressure, heart rate, and muscle fatigue and increases muscle oxygenation [6, 19–22], whereas others show activation of the sympathetic nervous system [23–25]. This inconsistency might be because of differences in massage technique, treatment time, or operator skill level which are not standardized between studies. Therefore, the need for systematic studies has been suggested [8, 26].

One way to overcome inconsistent outcomes with massage treatment is to use an automated massage device. The automated massage device has some advantages compared to the traditional massage methods performed by the therapist. First, it does not depend on the therapists' physical condition and always delivers a consistent pressure. Also, the massage pressure and location can be controlled precisely depending on the subjects' physical condition. Third, the automated massage can easily be combined with thermotherapy, where recent studies have suggested that massage accompanied with skin heating may have positive effects, such as increased skin blood flow [27] and decreased plasma cortisol and norepinephrine [28].

The current study evaluated the effect of automated massage therapy from three perspectives: physical, physiological, and psychological. We have used trunk extension (TE) and electromyography (EMG) as physical measures; heart-rate variability (HRV) and EEG as physiological measures; and state anxiety inventory (STAI-X-1) and visual analogue scale (VAS) as psychological measures. The massage therapy was controlled using an automatic chiropractic massage bed to minimize the performance of the massages. We also tested the effect of a combined massage program that consisted of pressure massage and thermotherapy.

## 2. Materials and Methods

### 2.1. Participants

Twenty-eight healthy participants (15 men and 13 women) were recruited through on-campus advertisements, bulletin boards, or verbal requests. An initial screening interview was conducted to check for history of psychiatric disease, mood disorder, brain injury, cardiovascular disease, or if he/she was on medications that might influence their response to treatment. The participants' demographic data are given in Table 1. All participants were right-handed and had normal or corrected-normal vision. The mean age of the male participants was 26.2 ± 2.68 years, and their average mean body mass index (BMI) was 24.69 ± 1.82; the mean age of the female participants was 23.26 ± 1.82 years, and their average BMI was 19.33 ± 1.34. There were statistical differences between genders in age (*p* = 0.009). All experimental procedures were approved by Yonsei University Wonju Campus Human Studies Committee (approval number: 2011-15).

### 2.2. Test Conditions and Procedure

**Figure 1:**
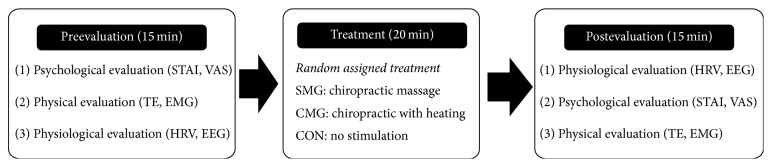
Overall procedure of the experiment. All subjects underwent 3 different treatment conditions in separate days (SM: single massage, CM: combined massage, and CON: control). The treatments were given to each participant in random order.

**Figure 2:**
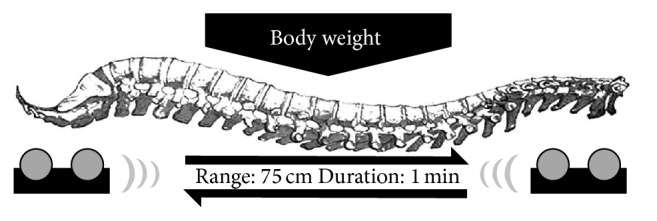
Schematic view of the automatic chiropractic massager. A pair of rollers massages the muscles along the spine by moving up and down from cervical vertebrae to coccygeal vertebra.

Upon enrollment, the participants were scheduled for 3 different visits in a week. During each visit, the participants were treated with either chiropractic (single massage [SM]), chiropractic with infrared heating stimulation (combined massage [CM]), or control (CON) treatment. For SM, the participants laid on an automatic spine massage bed (NM-5000; Nuga Medical, Wonju, Korea) and underwent a 20 min preprogrammed chiropractic massage sequence (Figure 1). In this sequence, a roller massages the muscles along the spine by moving up and down from cervical vertebrae to coccygeal vertebra (Figure 2). For CM, a heat source was added to the 20 min massage program. The heat was delivered using the heating light source located inside the roller. The temperature of the light source was set to 60°C (140°F). For CON, the participant laid on the massage bed without any massage/heat stimulation. All participants were asked to close their eyes, yet to keep themselves alert during the experiment.

### 2.3. Psychological, Physical, and Physiological Evaluations

#### 2.3.1. Physical Evaluation

To evaluate the physical changes before and after each treatment, EMG signals were measured during TE. The performer first lies prone on the floor and interlaces the fingers behind the head, which is the rest position. While the assistant secures the performer's hip against the floor, on the instruction “go,” the performer has to raise the chest and head from the floor as far as possible. TE was measured by the distance between the participants' chin and floor during trunk extension, indicating the flexibility of the trunk and also the fatigue and strength of the back muscle [29, 30]. The performance was measured by the average height of three attempts.

The EMG signal of erector spinae was recorded during TE, by attaching two electrodes of 4 cm horizontally centered on the participants' L3. The signal was recorded using EMG100C amplifier (Biopac Systems, Inc., USA) with sampling frequency of 1000 Hz. The EMG signal was filtered online using a 10–500 Hz band pass filter. Then, the root mean square (RMS) of the EMG signal during TE was averaged, which indicated the maximum strength of erector spinae muscles [31, 32].

#### 2.3.2. Physiological Evaluation

Physiological changes between pre- and posttreatment were evaluated using the ratio between high frequency and low frequency (LF/HF); heart rate (HR) derived from electrocardiogram (ECG); and spectral power of delta (1–4 Hz), theta (5–7 Hz), alpha (8–12 Hz), beta (13–30 Hz) band of EEG recordings. ECG and EEG were recorded for 5 min before and after the treatment. The participants were asked to lie on the massage bed and relax with their eyes closed to prevent any motor or ocular artifacts during measurement.

The ECG was measured using MP150 data acquisition system (Biopac Systems, Inc., USA) sampled at 1000 Hz with 0.5–35 Hz band pass filter. The recordings were done following the standard limb leads method. HR was acquired using R-R interval series. The LF/HF ratio was calculated by dividing the average power of high frequency component (0.15–4 Hz) to low frequency component (0.04–0.15 Hz) of the R-R interval series. The power spectrum was calculated using fast Fourier transform (FFT) with Hamming window applied to the whole data. All HRV measures were calculated using Acknowledge 4.1 (Biopac Systems, Inc., USA) Software.

EEG was measured using an EEG acquisition system (WEEG-32; Laxtha Inc., Daejeon, Korea). Scalp readings were recorded in 2 frontal lobe locations (F3 and F4) referenced at Cz [33]. The sampling frequency was 512 Hz with a 0.5–64 Hz band pass filter applied to the recording. The recordings were then divided into 2 s epochs and were visually inspected to reject any epochs with artifacts (i.e., muscle artifacts). The spectrum was calculated using FFT for the artifact-free epochs and the spectrum was averaged for each band (delta, theta, alpha, and beta). All analysis procedure was done using an in-house coded program with MATLAB 2009a (Mathworks, Inc., USA).

#### 2.3.3. Psychological Evaluation

Psychological changes before and after each treatment were evaluated using STAI and VAS. STAI is a self-report series of 20 items designed to evaluate state and trait anxiety in adults [34]. The level of stress was evaluated using VAS [35, 36]. The participants were provided with a paper with a 10 cm line, where the two ends of the line were marked to reflect extreme states of emotion. The participants were asked to report their current stress level by marking a spot on the line. The length between the left end of the line and the spot is proportional to stress.

## 3. Results

### 3.1. Physical Evaluation

**Figure 3:**
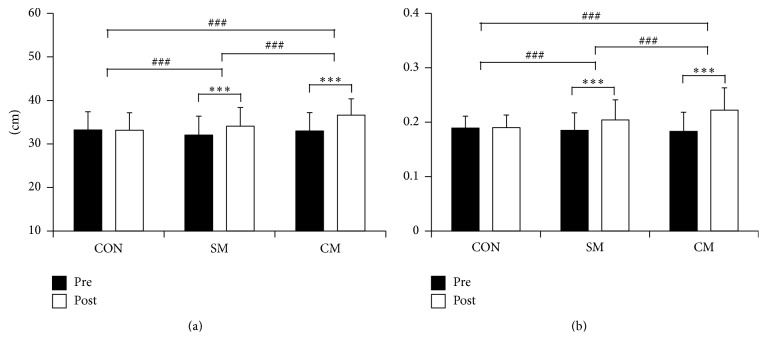
Physical changes of each treatment condition represented as (a) trunk extension (TE) and (b) electromyography root mean square (EMG-RMS) (CON: control, SM: single massage, and CM: combined massage; ^*∗*^ *p* < 0.05, ^*∗∗*^ *p* < 0.01, ^*∗∗∗*^ *p* < 0.001, ^#^ *p* < 0.05, ^##^ *p* < 0.01, ^###^ *p* < 0.001).

TE significantly differed after treatment in SM (pre versus post; 32.04 ± 4.32 versus 34.06 ± 4.31, *p* < 0.001) and CM condition (32.97 ± 4.22 versus 36.60 ± 3.75, *p* < 0.001) but not in CON condition (33.20 ± 4.21 versus 33.13 ± 4.03, *p* = 0.715; Figure 3(a)). Posttreatment height differed significantly between treatments (*F*(2) = 45.697, *p* < 0.001). Post hoc analysis revealed significant differences in all condition pairs: CON versus SM (*p* < 0.001), CON versus CM (*p* < 0.001), and SM versus CM (*p* < 0.001).

Changes in EMG-RMS showed similar trend to those in TE: SM and CM showed significant differences (SM: 0.185 ± 0.032 versus 0.204 ± 0.037, *p* < 0.001; CM: 0.183 ± 0.035 versus 0.222 ± 0.041, *p* < 0.001) but not in CON (0.189 ± 0.022 versus 0.190 ± 0.023, *p* = 0.675; Figure 3(b)). Differences between treatments were statistically significant (*F*(2) = 45.966, *p* < 0.001). Post hoc analysis showed differences in all condition pairs: CON versus SM (*p* < 0.001), CON versus CM (*p* < 0.001), and SM versus CM (*p* < 0.001).

### 3.2. Physiological Evaluation

**Figure 4:**
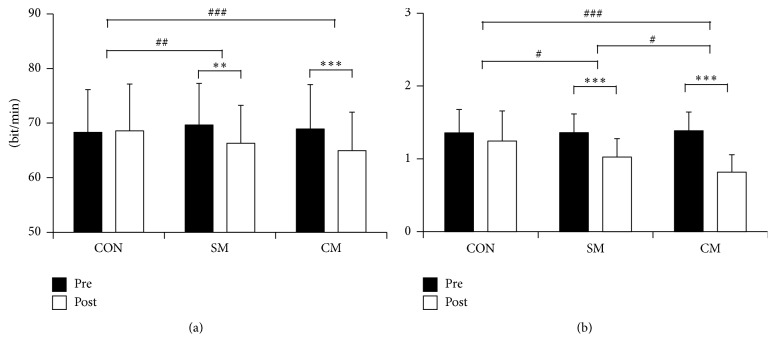
Physiological changes of each treatment condition in two heart-rate variability (HRV) related measures: (a) heart rate and (b) LF/HF ratio (CON: control, SM: single massage, and CM: combined massage; ^*∗*^ *p* < 0.05, ^*∗∗*^ *p* < 0.01, ^*∗∗∗*^ *p* < 0.001, ^#^ *p* < 0.05, ^##^ *p* < 0.01, ^###^ *p* < 0.001).

HR decreased significantly in SM and CM but not in CON (CON: 68.30 ± 7.97 versus 68.58 ± 8.74; SM: 69.65 ± 7.76 versus 66.29 ± 7.06, *p* = 0.003; CM: 68.93 ± 8.27 versus 64.95 ± 7.19, *p* < 0.001; Figure 4(a)). CON did not show significant change in HR between pre- and posttreatment (*p* = 0.645). The main effect on treatment was significant in HR (*F*(2) = 9.091, *p* < 0.001), where CON versus SM (*p* = 0.005) and CON versus CM (*p* < 0.001) showed significant changes in post hoc analysis. However, SM and CM did not show significant differences in posttreatment HR (*p* = 1.000).

Pre-post comparison between LF/HF ratio was significant in SM (1.36 ± 0.26 versus 1.02 ± 0.26, *p* < 0.001) and CM condition (1.38 ± 0.26 versus 0.82 ± 0.24, *p* < 0.001) but not in CON (1.36 ± 0.33 versus 1.24 ± 0.42, *p* = 0.157; Figure 4(b)). Significant differences in posttreatment LF/HF ratio were found (*F*(2) = 17.185, *p* < 0.001), and post hoc analysis showed significant differences between all conditions: CON versus SM (*p* = 0.013), CON versus CM (*p* < 0.001), and SM versus CM (*p* = 0.014).

**Figure 5:**
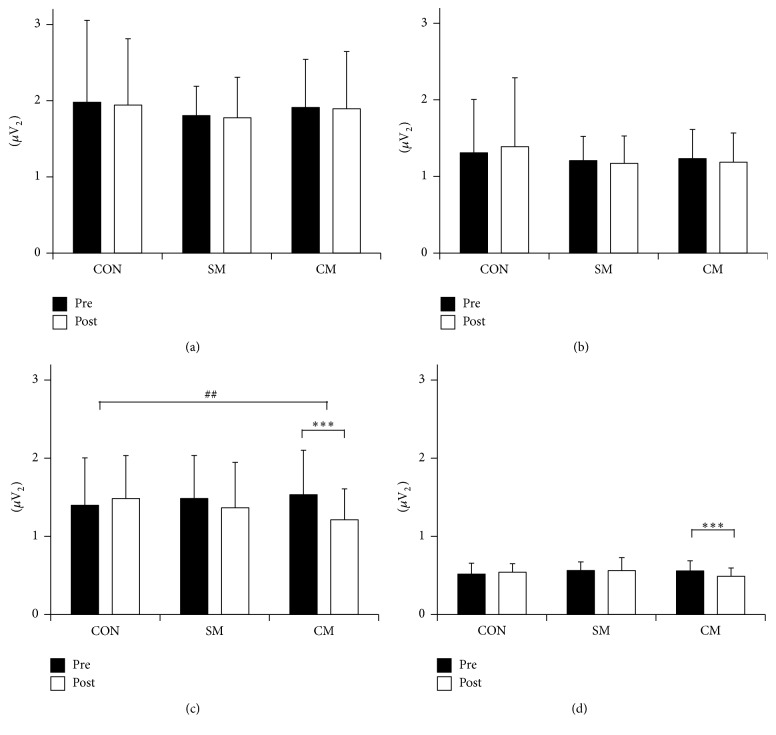
Physiological changes between pre- and posttreatment represented as power in four distinct frequency bands of EEG: (a) delta, (b) theta, (c) alpha, and (d) beta (CON: control, SM: single massage, and CM: combined massage; ^*∗*^ *p* < 0.05, ^*∗∗*^ *p* < 0.01, ^*∗∗∗*^ *p* < 0.001, ^#^ *p* < 0.05, ^##^ *p* < 0.01, ^###^ *p* < 0.001).

Differences in EEG power were significant only in alpha and beta band between pre- and posttreatment in CM (alpha: 1.533 ± 0.569 versus 1.212 ± 0.395, *p* = 0.004; beta: 0.558 ± 0.129 versus 0.489 ± 0.106, *p* = 0.007; Figure 5). Posttreatment alpha power was significantly different between treatments (*F*(2) = 5.853, *p* = 0.005), and post hoc analysis revealed significant differences between CON and CM (*p* = 0.003).

### 3.3. Psychological Evaluation

**Figure 6:**
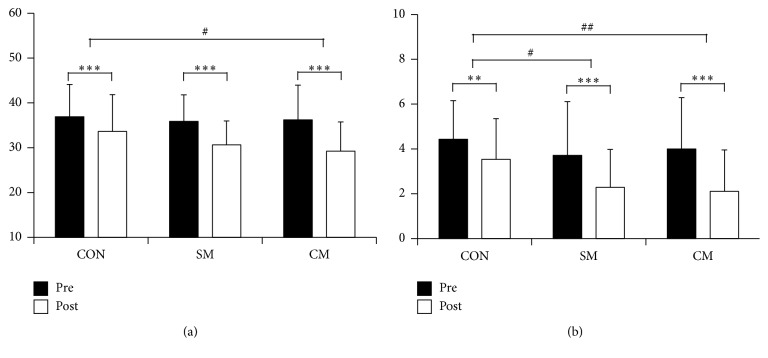
Psychological changes of each treatment condition represented as (a) state-trait anxiety inventory (STAI-X-1) and (b) visual analogue scale (VAS) (CON: control, SM: single massage, and CM: combined massage; ^*∗*^ *p* < 0.05, ^*∗∗*^ *p* < 0.01, ^*∗∗∗*^ *p* < 0.001, ^#^ *p* < 0.05, ^##^ *p* < 0.01, ^###^ *p* < 0.001).

Figure 6 represents the psychological changes of each treatment condition. All groups showed significant decrease in STAI-X-1 compared to pretreatment (CON: 36.89 ± 7.11 versus 33.61 ± 8.54, *p* = 0.008; SM: 35.86 ± 6.06 versus 30.61 ± 6.24, *p* < 0.001; CM: 36.21 ± 7.61 versus 29.21 ± 6.34, *p* < 0.001; Figure 6(a)). Posttreatment STAI-X-1 had a significant main effect in treatment (*F*(2) = 4.321, *p* = 0.017), and post hoc analysis revealed significant differences between CON and CM (*p* = 0.013).

VAS scores also showed significant decrease after each treatment (CON: 4.43 ± 1.67 versus 3.54 ± 1.77, *p* = 0.001; SM: 3.71 ± 2.31 versus 2.29 ± 1.67, *p* < 0.001; CM: 4.00 ± 2.24 versus 2.11 ± 1.79, *p* < 0.001; Figure 6(b)). These changes were significantly different between treatments (*F*(2) = 7.481, *p* = 0.001), with post hoc analysis revealing significant differences in posttreatment VAS scores between CON and SM (*p* = 0.034) and also between CON and CM (*p* = 0.001). However, there were no differences between SM and CM (*p* = 0.719).

## 4. Discussion

In the present study, we found that automated massage therapy has not only a significant physical effect, represented by increment of TE and EMG-RMS, but also a significant physiological effect, represented by decrement in HR, LF/HF ratio of HRV and reduced alpha and beta EEG power, and psychological effect, represented by decrement of STAI-X-1 and VAS scores. Most of the effects were more significant when massage therapy was combined with infrared heating.

Previous studies investigating the effects of massage therapy related to muscle characteristics have found positive effects such as increased muscle range of motion, maximum muscle strength, or flexibility [2, 26, 37]. For instance, Shambaugh [38] showed that pressure massage affects muscle recovery from physical stress or muscle fatigue, indicated as increased muscle flexibility and maximum muscle activation. Another previous study showed similar results of increased maximum strength and TE [21]. In the current study, both TE and EMG-RMS of SM and CM, but not of CON, were significantly increased after treatment. However, CM showed the most significant increment in both TE and EMG-RMS.

According to studies focusing on time domain analysis of HRV, massage therapy showed decreased stress response indicated as significantly decreased mean HR [3, 39, 40]. In the case of frequency domain analysis, massage therapy demonstrated an increase in HF leading to a decrease in LF/HF ratio [6]. Both mean HR and LF/HF ratio are associated with parasympathetic activity, with reduction of those values indicating an increase in parasympathetic activity [3, 6, 41–44]. Such increased parasympathetic activity owing to massage therapy appears to improve blood circulation through antagonistic activity of the autonomic nervous system, which helps to recover the physiological balance of tissues and organs of the human body [21, 45]. Therefore, the reduced LF/HF ratio or HR resulting from massage therapy alone seems to activate the parasympathetic nervous system, and adding infrared heating to massage therapy might increase the activation. Another physiological factor, EEG, showed decreased frontal alpha and beta band power between pre- and posttreatment, but only alpha band showed significant differences between treatment conditions. The results are coherent with studies using EEG to investigate the effect of moderate massage, which usually decreased frontal alpha and beta band power while increasing delta power, suggesting a relaxation response [3].

With regard to the psychological aspects, STAI and VAS were used to determine the change of participants' anxiety and stress level before and after the massage therapy. The results indicated that anxiety and stress are decreased regardless of treatment condition, which is consistent with previous reports of decreased STAI and VAS after pressure massage [46–48]. However, the posttreatment scores showed significant reduction in treatment condition, which revealed that anxiety and stress are more efficiently relieved by massage therapy with infrared heating than by massage therapy alone.

Integrating the results together, the current result supports the hypothesis that massage therapy activates the parasympathetic nervous system, thus leading the body to relax represented by physical, physiological, and psychological responses. Furthermore, we have revealed that the combination of infrared heating with massage therapy was more beneficial in terms of relaxation by more significant responses of the body.

Previous studies have evaluated the effectiveness of massage performed by a masseur. However, massage by the masseur might be a source of human errors [49]. In this study, it was possible to minimize human errors by employing uniform, automated massage for all participants. Through this experimental control, we considered to get a more clear assessment of the efficacy of massage.

The present study has some limitations. First, the measures used in this study were indirect measures for evaluation of the balance of the autonomic nervous system; more direct measurements related to parasympathetic/sympathetic might be preferable. For instance, biochemical measures such as cortisol [42] or oxytocin level [50] are hormonal responses related to the balance of the autonomic nervous system and also immune interactions. Especially, the cortisol level is able to estimate the hypothalamic-pituitary-adrenal axis which is another neural stress system [51, 52]. Second, the ages of participants of this study were limited. There are evidences that EEG [53] or HRV [54] vary according to age; thus, it would be necessary to investigate if the effect of the massage is consistent across ages.

## 5. Conclusion

In the current study, we observed that massage therapy contributed to psychological stability, improved trunk flexibility, maximal strength of the muscle, and activating parasympathetic nerves. When massage was combined with infrared heating, a more effective response was observed. Hence, the combination of pressure and heating massage could be offered as an alternative treatment method to help to prevent musculoskeletal pain and to relieve stress.

## Figures and Tables

**Table 1:** Demographic data of the participants.

Male : female	15 : 13
Age (years)	25.00 ± 2.66
Weight (kg)	64.46 ± 12.92
Height (cm)	168.29 ± 7.68
BMI	22.20 ± 3.15

BMI: body mass index.
